# Cost‐effective immobilization for whole brain radiation therapy

**DOI:** 10.1002/acm2.12101

**Published:** 2017-06-06

**Authors:** Ashley E. Rubinstein, W. Scott Ingram, Brian M. Anderson, Skylar S. Gay, Xenia J. Fave, Rachel B. Ger, Rachel E. McCarroll, Constance A. Owens, Tucker J. Netherton, Kelly D. Kisling, Laurence E. Court, Jinzhong Yang, Yuting Li, Joonsang Lee, Dennis S. Mackin, Carlos E. Cardenas

**Affiliations:** ^1^ Department of Radiation Physics The University of Texas MD Anderson Cancer Center Houston TX USA; ^2^ Graduate School of Biomedical Sciences The University of Texas Health Sciences Center Houston TX USA

**Keywords:** accuracy, immobilization, low‐ and middle‐income countries, palliation, setup, whole‐brain treatment

## Abstract

To investigate the inter‐ and intra‐fraction motion associated with the use of a low‐cost tape immobilization technique as an alternative to thermoplastic immobilization masks for whole‐brain treatments. The results of this study may be of interest to clinical staff with severely limited resources (e.g., in low‐income countries) and also when treating patients who cannot tolerate standard immobilization masks. Setup reproducibility of eight healthy volunteers was assessed for two different immobilization techniques. (a) One strip of tape was placed across the volunteer's forehead and attached to the sides of the treatment table. (b) A second strip was added to the first, under the chin, and secured to the table above the volunteer's head. After initial positioning, anterior and lateral photographs were acquired. Volunteers were positioned five times with each technique to allow calculation of inter‐fraction reproducibility measurements. To estimate intra‐fraction reproducibility, 5‐minute anterior and lateral videos were taken for each technique per volunteer. An in‐house software was used to analyze the photos and videos to assess setup reproducibility. The maximum intra‐fraction displacement for all volunteers was 2.8 mm. Intra‐fraction motion increased with time on table. The maximum inter‐fraction range of positions for all volunteers was 5.4 mm. The magnitude of inter‐fraction and intra‐fraction motion found using the “1‐strip” and “2‐strip” tape immobilization techniques was comparable to motion restrictions provided by a thermoplastic mask for whole‐brain radiotherapy. The results suggest that tape‐based immobilization techniques represent an economical and useful alternative to the thermoplastic mask.

## 1 INTRODUCTION

Whole‐brain radiation therapy (WBRT) is a common treatment for patients with advanced brain cancer and brain metastases. Patients are typically immobilized in the supine position with a thermoplastic mask and treated with parallel opposed lateral fields.^1^ The delivery of WBRT has been shown to improve intracranial control in patients with multiple brain metastases^2^, ^3^, ^4^ and remains an integral part of palliative treatment for advanced stage metastatic brain cancer patients.

In many institutions, the current standard of care for WBRT uses thermoplastic masks to reduce inter‐ and intra‐fraction patient positioning uncertainties.^5^ Advances in radiotherapy have led to many sophisticated solutions for head and neck immobilization.^6^, ^7^, ^8^, ^9^, ^10^ However, in resource‐limited settings, including some low‐ and middle‐income countries, these immobilization solutions may not be widely available for clinical use. Thermoplastic masks can be expensive and, in developed countries, are typically discarded after each patient completes his or her treatment. When resources are limited, these masks can be reused for multiple patients, although they generally degrade after two to three patients. Therefore, other immobilization techniques such as tape and rice bags are sometimes used for treatment immobilization.^11^ This setup is also currently used for emergency treatments in some centers. In addition, some patients find the mask very difficult to tolerate and could benefit from a less restrictive immobilization technique.

Several studies have investigated positioning uncertainty for stereotactic radiosurgery^6^, ^7^, ^8^, ^9^, ^10^, ^12^ and head and neck treatments.^13^, ^14^, ^15^, ^16^ These studies used a variety of approaches to assess inter‐ or intra‐fraction motions, including orthogonal x‐ray imaging, cone‐beam computed tomography, and ultrasound.^7^, ^17^, ^18^, ^19^, ^20^ Using CBCT evaluation, Lightstone et al.^19^ showed an inter‐fraction uncertainty of 2.9 mm (L‐R: 1.6 mm, A‐P: 1.7 mm, S‐I: 1.1 mm) and intra‐fraction uncertainty of 0.76 mm (L‐R: 0.29 mm, A‐P: 0.54 mm, S‐I: 0.25 mm). Ojerolm et al. showed that the inter‐fraction uncertainty during WBRT is in the magnitude of 1 mm, 1 mm, and 2 mm in the L‐R, A‐P, and S‐I directions, respectively.^18^ Other studies have reported the same magnitude of inter‐ and intra‐fractional uncertainties for WBRT.^21^ When using thermoplastic masks for patient immobilization, inter‐ and intra‐fraction uncertainty has been reported to be between 1 and 3 mm.^13^, ^14^, ^15^, ^16^ In another study using optical 3D surface imaging to assess intra‐fraction motion for whole‐brain treatments, Wiant et al. found that the average position change was submillimeter in magnitude using open face thermoplastic masks.^22^

There are few studies on simple immobilization techniques. One study, published during the transition to thermoplastic mask immobilization systems, quantified the number of isocenter shifts. It showed a reduction in repositioning frequency when thermoplastic masks were fixed to the treatment table compared to when straps were used.^23^ However, they did not report the magnitude of positioning errors and did not assess intra‐fraction uncertainties. The assessment of the reproducibility of simple, tape‐based immobilization technique for whole‐brain treatments would allow clinician confidence in using these techniques and could guide the development of accurate margins for treatment.

In this study, the viability of a simple method for WBRT immobilization was evaluated. Optical imaging was used to assess inter‐ and intra‐fraction reproducibility of two techniques, and these were compared to published values of mask‐based techniques. This study represents an addition to the published literature allowing for confidence in the use of a simple, low‐cost, and effective immobilization technique for WBRT.

## 2 MATERIALS AND METHODS

### 2.A Treatment setup

**Figure 1:**
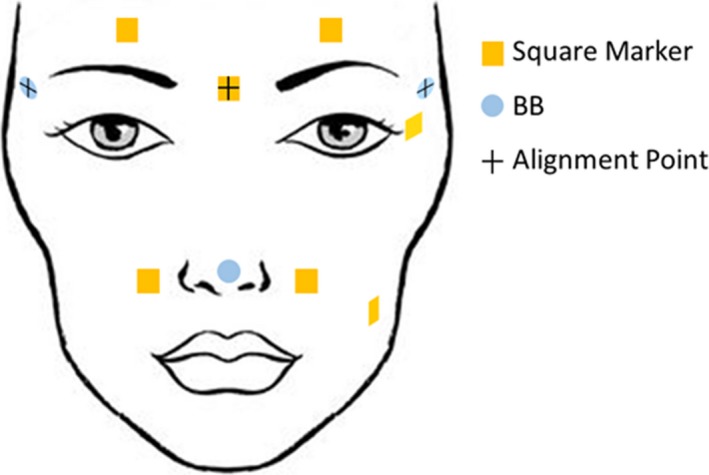
Illustration of marker and BB locations. The square marker and BBs with crosses were used for setup.

This study was approved by our local institutional review board and consent was acquired for each volunteer. Eight healthy volunteers (median age: 27, three males and five females) were positioned on the treatment table of a clinical linear accelerator (Varian Medical Systems, Palo Alto, CA, USA) using two different immobilization methods. Each volunteer was positioned on the treatment table lying supine with their head on a Silverman headrest (Civco Radiotherapy, Coralville, IA, USA) size A or C, whichever was most comfortable. The headrest was indexed to the table. Square markers of a fixed size (0.5 inch) were placed on the volunteer's head to track their motion, and BBs were placed to align the volunteer with the in‐room lasers (Fig. 1).

**Figure 2:**
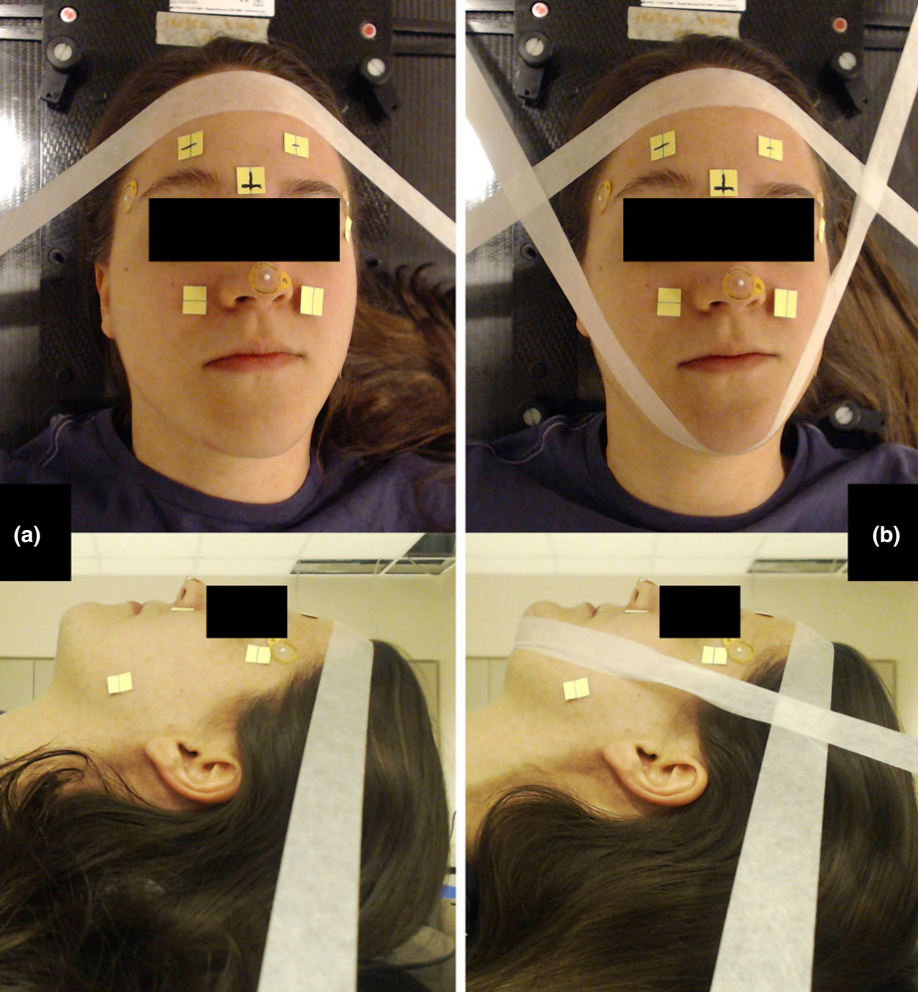
Volunteer setup. Volunteers were immobilized using a 1‐strip (panel a) and 2‐strip technique (panel b). Anterior (above) and lateral (below) photographs and videos were taken of the volunteers to assess inter‐ and intra‐fractional reproducibility.

Two immobilization techniques were compared in this study. In the first method (“1‐strip technique”), a strip of surgical tape was placed across the volunteer's forehead and attached to the sides of the table slightly inferior to the alignment point [Fig. 2(a)]. In the second method (“2‐strip technique”), a second strip of surgical tape was added under the chin and attached to the superior edge of the table [Fig. 2(b)]. All positioning was performed by the same two physicists, both of whom have clinical experience.

Two orthogonal 15 megapixel cameras (Logitech C920, Newark, CA, USA) were positioned in the room. One was attached to the accessory tray on the gantry head with the gantry at 0° to acquire an anterior video with 1080p at 30 frames/sec. The second camera was set up on a tripod fixed to the floor to acquire a lateral video from the volunteer's left side. Anterior and lateral static images were acquired after each volunteer was positioned on the treatment table. Once the pictures were collected, the patient left the treatment table, and the next volunteer was set up. This was repeated to simulate multiple fractions. Five sets of images (five fractions) were collected per volunteer and per immobilization technique to evaluate inter‐fraction variability in setup, alternating volunteers between sets of images. In addition, each volunteer was continuously video‐recorded for 5 minutes to evaluate intra‐fraction motion for each immobilization technique.

### 2.B Description of software

In‐house software^24^ was used to evaluate intra‐fraction motion of the square markers. The anterior video was used to calculate intra‐fraction motion in the superior–inferior (S‐I) and left–right (L‐R) directions. The corners of the central square marker were manually selected and used to calibrate the pixel size in the image. Corner detection and optical flow were used to track the marker motion throughout the 5‐minute video. For the lateral video, the superior square marker (closest to the temple) was used to calibrate the pixel size, and intra‐fraction motion was calculated for the S‐I and anterior–posterior (A‐P) directions.

In the five inter‐fraction images, the corners of one square marker were manually selected and used to calibrate the pixel size in the image. The central marker was used to calibrate the anterior images, and the inferior marker was used to calibrate the lateral images. To quantify inter‐fraction motion, one corner of a square marker was manually selected, and the range of its positions was measured in each of the five images. For the anterior view, the left superior marker was used to measure setup reproducibility in the S‐I and L‐R directions. For the lateral view, the superior marker (temple) was used to measure setup reproducibility in the S‐I and A‐P directions.

The software's ability to accurately calculate motion was validated using a video with a marker moving a known distance. In the validation video, a square marker was taped on the treatment table, and the table was shifted 10 mm manually in both longitudinal and lateral directions. Since manual selection of marker corners is required for pixel size calibration of the software, the video with the known shift was measured 10 times to test the manual selection consistency.

### 2.C Analysis of inter‐ and intra‐fraction motion

For the intra‐fraction measurements made using both the anterior and lateral cameras, paired samples t‐tests were used to compare the difference between the two immobilization methods. Six t‐tests were conducted to determine if the difference between the range of positions was significant for the two immobilization techniques: (a) anterior camera view at 3 min, (b) anterior camera view at 5 min, (c) maximum displacement in anterior camera view, (d) lateral camera view at 3 min, (e) lateral camera view at 5 min, and (f) maximum displacement in lateral camera view.

For the inter‐fraction study, the range of positions in the x and y directions in the image was obtained for each volunteer for both setup techniques and both cameras. For the anterior camera, x direction refers to S‐I direction and y direction refers to medial‐lateral direction. For the lateral camera, x direction refers to S‐I direction and y direction refers to A‐P direction. Four t‐tests were conducted to determine if the difference between the range of positions was significant for the two immobilization techniques: (a) anterior camera view of the x direction displacement, (b) anterior camera view of the y direction displacement, (c) lateral camera view of the x direction displacement, and (d) lateral camera view of the y direction displacement.

## 3 RESULTS

For the marker motion validation, the mean shift measured by the software for a 10.0 mm manual shift was 9.9 ± 0.1 mm along the lateral direction and 9.6 ± 0.1 mm along the longitudinal direction. Overall, the uncertainty in any single measurement would be within 0.5 mm.

**Figure 3:**
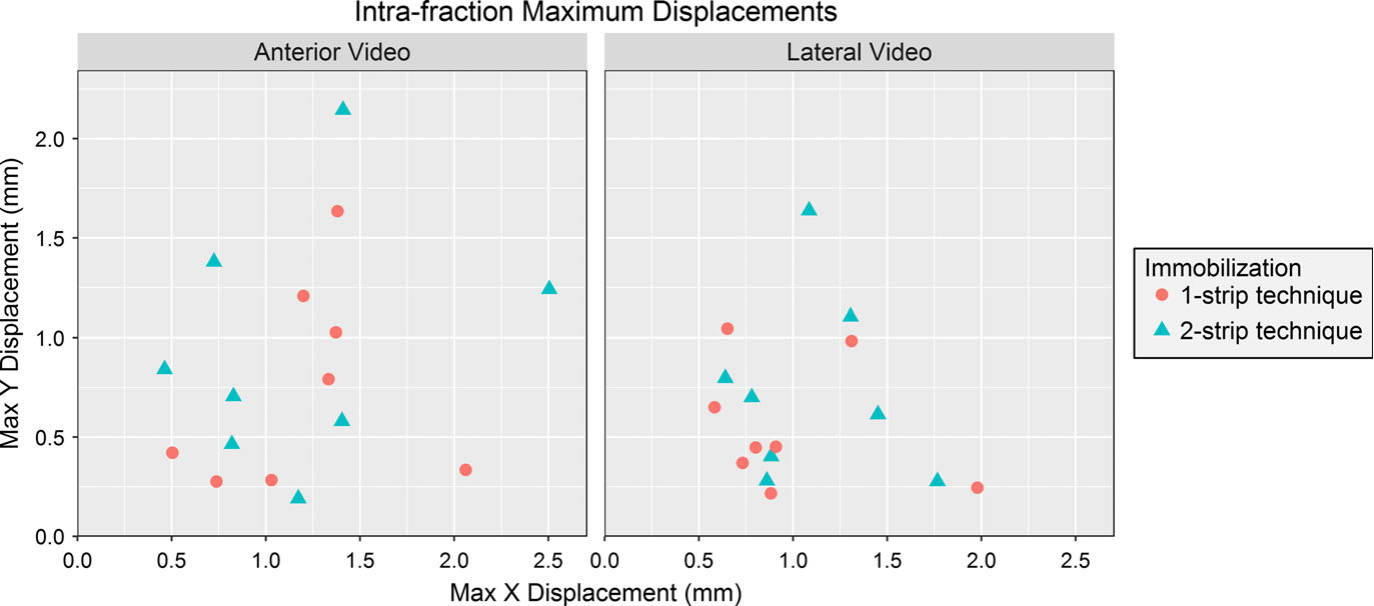
The maximum intra‐fraction displacements. In the x and y directions for the 1 and 2 strip immobilization techniques for the lateral (left) and anterior cameras (right).

**Figure 4:**
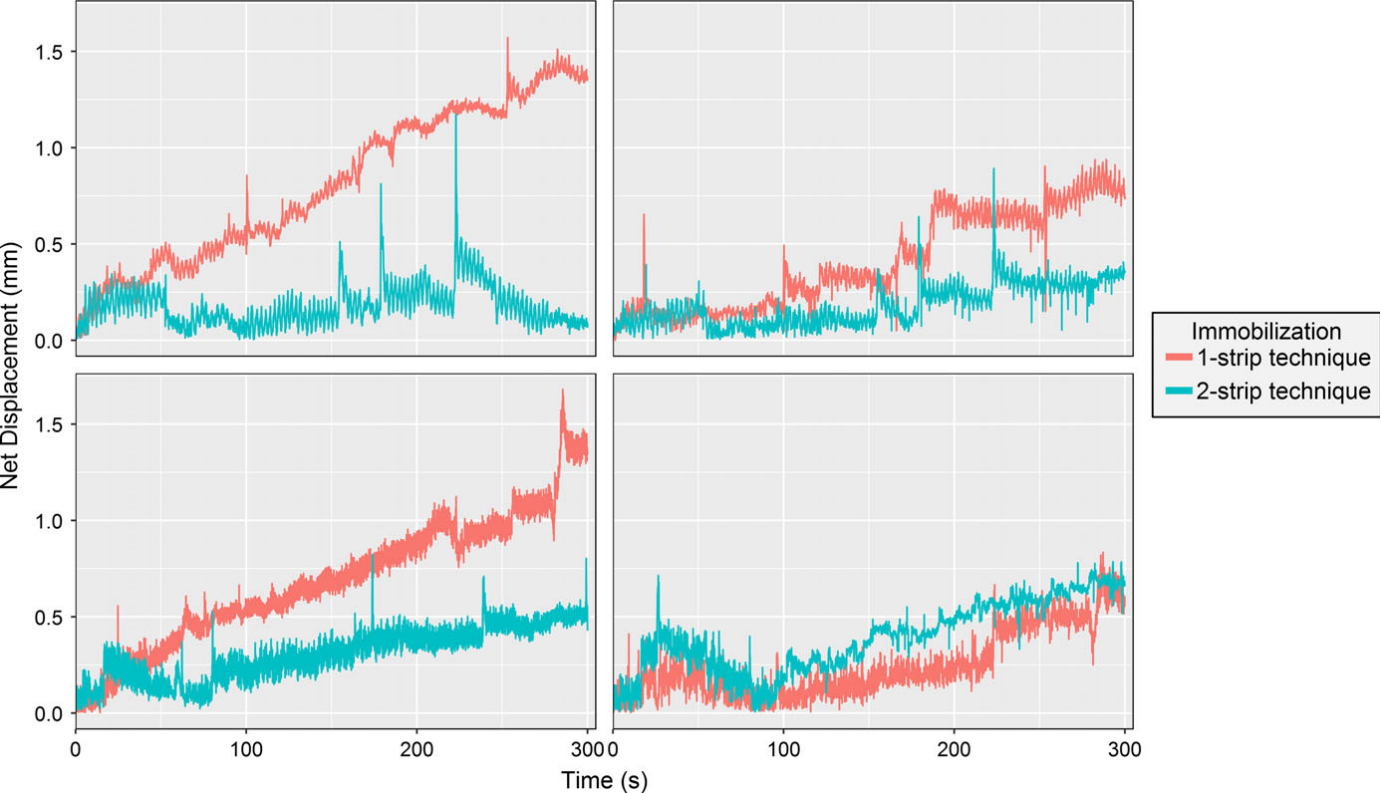
Comparison of the intra‐fraction motion for two immobilization techniques. The data shown are for two volunteers using the video from the lateral and anterior camera. The Euclidean (net) displacement was calculated and plotted for each frame of the video. Graphs for all volunteers and all camera views are available in the Supplementary Data.

Figure 3 shows the maximum intra‐fraction displacement in the x and y directions for each volunteer for the two setup techniques. The maximum displacement for all volunteers was 2.8 mm. The intra‐fraction net displacements with time are shown for two volunteers in Fig. 4. It was observed that the displacement tends to increase with time, with the maximum displacement typically found at the end of the treatment. Displacement for all volunteers with time from the anterior and lateral cameras is in the Supplementary Data. Spikes were observed in the data and were due to sudden movements such as swallowing. None of the t‐tests comparing the immobilization methods were significant (Table 1).

**Table 1:** The Welch's *t*‐test results. Results of M1 and M2 for both anterior and lateral cameras for intra‐fraction motion. The data taken at 180 s and 300 s of each patient and the maximum displacement among all patients were analyzed

Data type	At 3 min			At 5 min			Maximum value		
	1‐Strip	2‐Strip	*P*‐value	1‐Strip	2‐Strip	*P*‐value	1‐Strip	2‐Strip	*P*‐value
	Mean (mm)	Mean (mm)		Mean (mm)	Mean (mm)		Mean (mm)	Mean (mm)	
Anterior camera	0.7 ± 0.4	0.8 ± 0.5	0.63	1.2 ± 0.7	1.2 ± 0.9	0.89	1.4 ± 0.6	1.5 ± 0.7	0.74
Lateral camera	0.5 ± 0.4	0.6 ± 0.4	0.36	0.9 ± 0.5	0.9 ± 0.5	0.54	1.1 ± 0.4	1.3 ± 0.4	0.10

**Figure 5:**
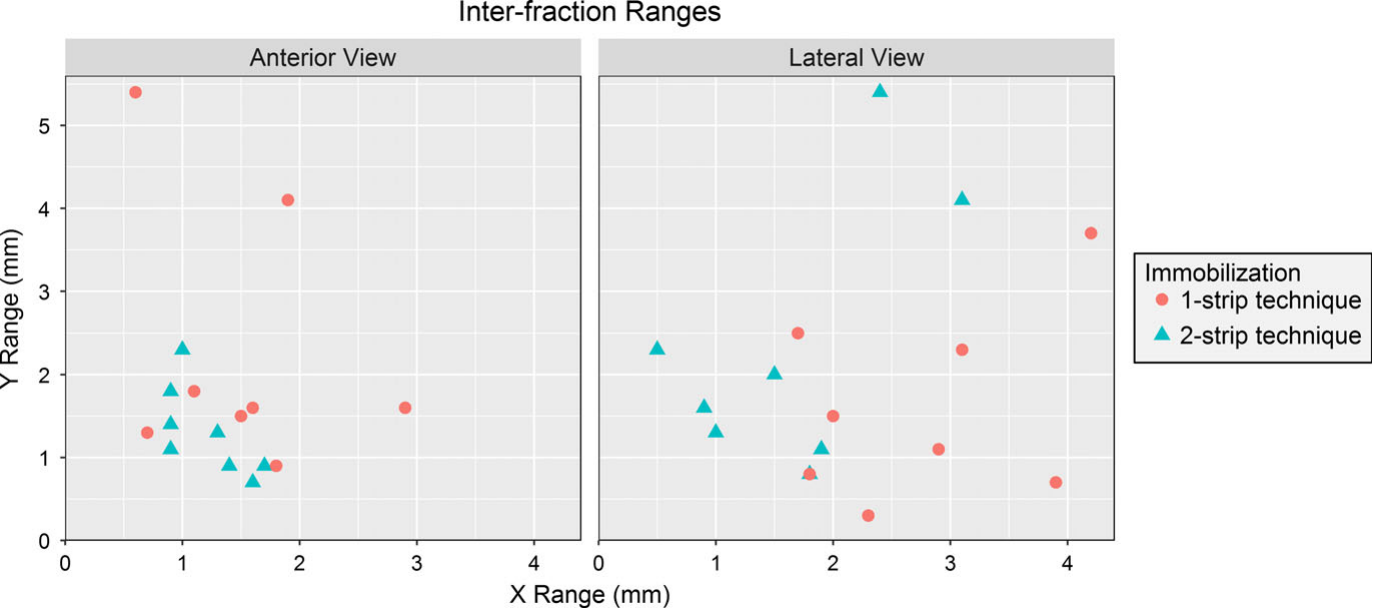
Maximum X and Y Inter‐fractional Displacement. The maximum inter‐fraction displacements in the x and y directions for the 1 and 2 strip immobilization techniques for the lateral (left) and anterior cameras (right).

Figure 5 shows the inter‐fraction range of positions in the x and y directions for each volunteer for the two setup techniques. The maximum range for all volunteers was 5.4 mm. The difference between the immobilization techniques for the lateral view in the x direction (S‐I) was significant (*P* < 0.05); however, the difference between the means was only 1 mm. All other comparisons were not significantly different (Table 2).

**Table 2:** The Welch's *t*‐test results. Results comparing the inter‐fraction ranges of the two techniques for both camera views

Camera		1‐Strip	2‐Strip	*P*‐value
		Mean (mm)	Mean (mm)	
Anterior camera	Range X	1.5 ± 0.7	1.2 ± 0.3	0.255
	Range Y	2.3 ± 1.6	1.3 ± 0.5	0.194
Lateral camera	Range X	2.7 ± 1.0	1.7 ± 0.8	0.008
	Range Y	1.6 ± 1.2	2.3 ± 1.6	0.253

## 4 DISCUSSION

Individual thermoplastic masks are widely used for localization and immobilization during radiation therapy of targets in the head region. Although necessary for complex head and neck treatments, these masks are also used for simpler treatments such as whole‐brain irradiation. The motivation for this study was to provide an initial evaluation of whether tape‐based immobilization techniques can provide adequate levels of immobilization for WBRT. If successful, this could lead to cost‐savings for these treatments which may be of particular interest to clinical professionals in resource‐limited settings.

When tape was used to immobilize the volunteers, the inter‐fraction range had a maximum magnitude of 5.4 mm and the intra‐fraction motion had a maximum magnitude of 2.4 mm. The two techniques had comparable intra‐fraction motion. All of the distance magnitudes were consistent with data reported for immobilization with commercial thermoplastic masks. It should be noted that methods to assess uncertainties vary widely so direct comparisons are not straightforward.

While thermoplastic masks have become the standard for reducing inter‐ and intra‐fraction position uncertainty for head and neck treatments and WBRT, this study shows that immobilization techniques using strips of tape may be reasonable alternatives for WBRT and could be used as surrogates in emergency procedures and limited resource environments. In addition, the tape immobilization techniques may be appropriate for patients suffering from claustrophobia while using the thermoplastic mask. One potential advantage of thermoplastic masks over the tape methods is the prevention of large intra‐fraction motion resulting from sudden movements such as coughing and sneezing. Such motion has been investigated in other studies.^17^

There are a few limitations to this study. First, the use of young and healthy volunteers to assess patient positioning may not reflect clinical scenarios with real patients. In addition, the intra‐fraction motion analysis might have not accurately represented a real treatment delivery because there was no motion of the gantry, collimator, or MLC which could distract or startle the patient. Lastly, only the shift (3D correction) without including the rotation (6D correction) was studied. Thermoplastic masks may have less uncertainty in 3D rotation, in particular, for inter‐fractional uncertainty, in comparison with the tape immobilization. As such, the results of this study probably represent a best‐case scenario.

The small magnitude of intra‐fraction motion found in this study is of particular interest to clinics where daily imaging is used to position patients. Although daily image‐guided radiation therapy (IGRT) is not standard practice when treating whole‐brain patients, it is not difficult to imagine a future where all patients are treated using daily IGRT given the overall gains in patient safety (such as eliminating patient setup or shift errors)^25^, ^26^, ^27^ that may be attributed to daily IGRT. Daily imaging could effectively minimize the inter‐fraction position uncertainties such that the intra‐fraction motion would become the main source of geometric uncertainty. Intra‐fraction motion could be even further reduced by limiting patient time on the treatment table as our results indicate that intra‐fraction motion tends to be a sliding shift, rather than random motion.^21^, ^28^

## 5 CONCLUSIONS

The magnitude of inter‐fraction and intra‐fraction motion found using the “1‐strip” and “2‐strip” tape immobilization techniques was comparable to motion restrictions provided by a thermoplastic mask for WBRT. The results suggest that tape‐based immobilization techniques have potential for treating whole‐brain patients. This is especially true if daily imaging is used to minimize inter‐fraction motion and treatment time is kept short to minimize intra‐fraction motion. Various limitations of this study mean that a patient study is needed before concrete treatment proposals can be developed.

## ACKNOWLEDGMENT

The authors thank the volunteers for their time and cooperation throughout this study.

## CONFLICT OF INTEREST

The authors declare no conflicts of interest.

## Supporting information

Fig. S1 Intra‐fractional data for all eight volunteers from both the anterior and lateral camera view using two immobilization techniques. Net displacements are calculated using the distance formula and the delta x and delta y values at each time point of the video. All net displacements were below 2.8 mm for both immobilization techniques.
Fig. S2 The maximum intra‐fractional displacement for each volunteer is plotted for each combination of immobilization technique and camera view.
Fig. S3 The inter‐fractional range for each volunteer is plotted for each combination of immobilization technique and camera view.
Table S1 The maximum displacement from first fraction along x and y directions captured by both cameras for intra‐fraction reproducibility.
Table S2 The range of values in the x and y directions by both cameras for inter‐fraction setup.
