# Supplementary material for: Cost‐effective immobilization for whole brain radiation therapy
Source: J Appl Clin Med Phys. 2017 Jun 6;18(4):116–22. doi: 10.1002/acm2.12101 (PMC5874864; doi:10.1002/acm2.12101)
Supplement: Supplementary file 1 — Fig. S1 Intra‐fractional data for all eight volunteers from both the anterior and lateral camera view using two immobilization techniques. Net displacements are calculated using the distance formula and the delta x and delta y values at each time point of the video. All net displacements were below 2.8 mm for both immobilization techniques. Fig. S2 The maximum intra‐fractional displacement for each volunteer is plotted for each combination of immobilization technique and camera view. Fig. S3 The inter‐fractional range for each volunteer is plotted for each combination of immobilization technique and camera view. Table S1 The maximum displacement from first fraction along x and y directions captured by both cameras for intra‐fraction reproducibility. Table S2 The range of values in the x and y directions by both cameras for inter‐fraction setup. [file ACM2-18-116-s001.docx]

**Supplementary Data**


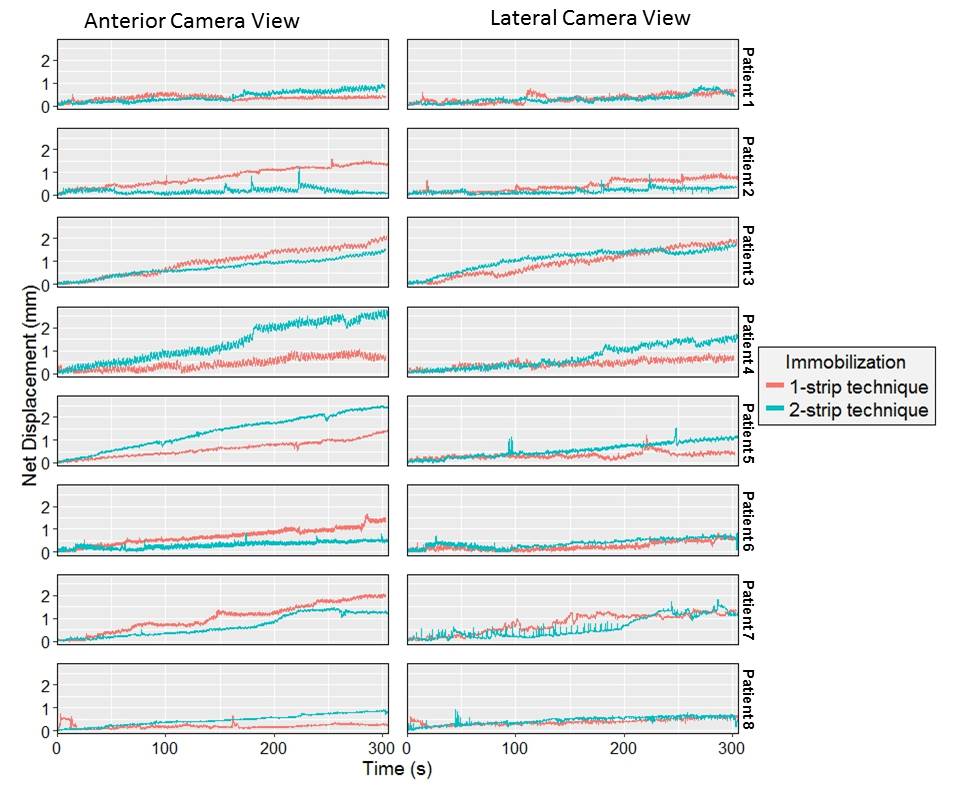


**Fig. S1**:Intra-fractional data for all eight volunteers from both the anterior and lateral camera view using two immobilization techniques. Net displacements are calculated using the distance formula and the delta x and delta y values at each time point of the video. All net displacements were below 2.8 mm for both immobilization techniques


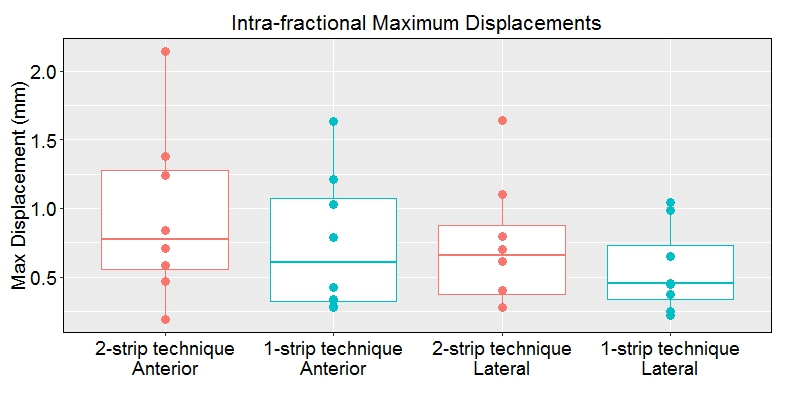


**Fig. S2**: The maximum intra-fractional displacement for each volunteer is plotted for each combination of immobilization technique and camera view.


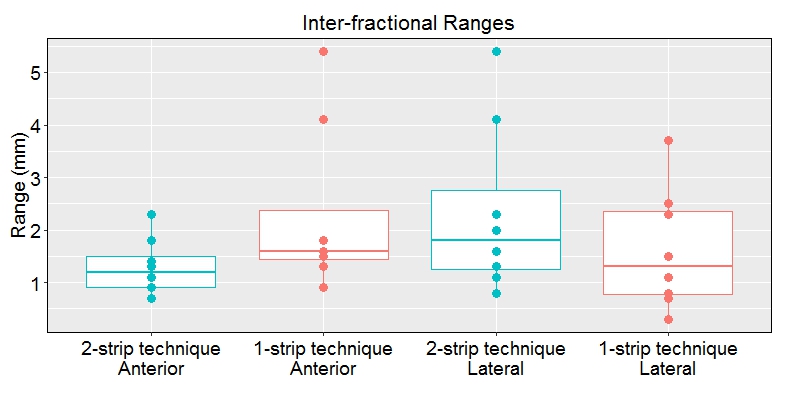


**Fig S3**: The  inter-fractional range for each volunteer is plotted for each combination of immobilization technique and camera view.

**Table S1: The maximum displacement from first fraction along x and y directions captured by both cameras for intra-fraction reproducibility.**

| Volunteer | Setup Method | Anterior Camera (mm) | | Lateral Camera (mm) | |
| --- | --- | --- | --- | --- | --- |
|  |  | max ∆Y | max ∆X | max ∆Y | max ∆X |
|  |  |  |  |  |  |
| 1 | 2-Strip | 0.7 | 0.8 | 0.3 | 0.1 |
|  | 1-Strip | 0.0 | 0.4 | 0.4 | 0.6 |
| 2 | 2-Strip | 0.2 | 0.3 | 0.4 | 0.9 |
|  | 1-Strip | 0.0 | 0.4 | 0.5 | 0.9 |
| 3 | 2-Strip | 0.0 | 0.1 | 0.1 | 1.8 |
|  | 1-Strip | 0.1 | 0.2 | 0.1 | 2.0 |
| 4 | 2-Strip | 0.0 | 2.5 | 0.1 | 0.1 |
|  | 1-Strip | 0.1 | 1.0 | 0.2 | 0.2 |
| 5 | 2-Strip | 0.0 | 1.4 | 0.1 | 0.1 |
|  | 1-Strip | 0.1 | 1.3 | 0.0 | 0.7 |
| 6 | 2-Strip | 0.0 | 0.4 | 0.8 | 0.4 |
|  | 1-Strip | 1.0 | 0.1 | 0.4 | 0.4 |
| 7 | 2-Strip | 0.0 | 0.4 | 0.1 | 1.1 |
|  | 1-Strip | 0.3 | 0.1 | 0.0 | 1.3 |
| 8 | 2-Strip | 0.0 | 0.2 | 0.7 | 0.2 |
|  | 1-Strip | 0.1 | 0.7 | 0.6 | 0.6 |

**Table S2: The range of values in the x and y directions by both cameras for inter-fraction setup.**

| Volunteer | Setup Method | Anterior Camera (mm) | | Lateral Camera (mm) | |
| --- | --- | --- | --- | --- | --- |
|  |  | Y range | X range | Y range | X range |
|  |  |  |  |  |  |
| 1 | 2-Strip | 0.9 | 1.7 | 4.1 | 3.1 |
|  | 1-Strip | 1.6 | 2.9 | 3.7 | 4.2 |
| 2 | 2-Strip | 0.7 | 1.6 | 1.1 | 1.9 |
|  | 1-Strip | 5.4 | 0.6 | 2.3 | 3.1 |
| 3 | 2-Strip | 1.1 | 0.9 | 0.8 | 1.8 |
|  | 1-Strip | 1.3 | 0.7 | 0.3 | 2.3 |
| 4 | 2-Strip | 0.9 | 1.4 | 2.0 | 1.5 |
|  | 1-Strip | 1.6 | 1.6 | 2.5 | 1.7 |
| 5 | 2-Strip | 1.3 | 1.3 | 1.3 | 1.0 |
|  | 1-Strip | 4.1 | 1.9 | 1.5 | 2.0 |
| 6 | 2-Strip | 1.8 | 0.9 | 1.6 | 0.9 |
|  | 1-Strip | 0.9 | 1.8 | 0.7 | 3.9 |
| 7 | 2-Strip | 2.3 | 1.0 | 5.4 | 2.4 |
|  | 1-Strip | 1.5 | 1.5 | 1.1 | 2.9 |
| 8 | 2-Strip | 1.4 | 0.9 | 2.3 | 0.5 |
|  | 1-Strip | 1.8 | 1.1 | 0.8 | 1.8 |
